# QTL mapping reveals candidate genes for main agronomic traits in *Luffa* based on a high-resolution genetic map

**DOI:** 10.3389/fpls.2022.1069618

**Published:** 2022-11-18

**Authors:** Lili Liu, Yaqin Gan, Jianning Luo, Junxing Li, Xiaoming Zheng, Hao Gong, Xiaoxi Liu, Liting Deng, Gangjun Zhao, Haibin Wu

**Affiliations:** ^1^ Guangdong Key Laboratory for New Technology Research of Vegetables, Vegetable Research Institute, Guangdong Academy of Agricultural Sciences, Guangzhou, China; ^2^ College of Coastal Agricultural Sciences, Guangdong Ocean University, Zhanjiang, China; ^3^ Guangdong Laboratory for Lingnan Modern Agriculture, Guangzhou, China

**Keywords:** *Luffa*, genetic linkage map, vital agronomic traits, QTL mapping, candidate genes, haplotype analysis

## Abstract

*Luffa* is an important medicinal and edible vegetable crop of Cucurbitaceae. Strong heterosis effects and strikingly complementary characteristics were found between the two domesticated *Luffa* cultivars, *Luffa acutangula* and *Luffa cylindrica*. To explore the genetic basis underlying their important agronomic traits, we constructed the first interspecific high-density genetic linkage map using a BC_1_ population of 110 lines derived from a cross between S1174 (*Luffa acutangula*) and P93075 (*Luffa cylindrica*). The map spanned a total of 2246.74 cM with an average distance of 0.48 cM between adjacent markers. Thereafter, a large-scale field-based quantitative trait loci (QTLs) mapping was conducted for 25 important agronomic traits and 40 significant genetic loci distributed across 11 chromosomes were detected. Notably, a vital QTL (*qID2*) located on chromosome 9 with a minimum distance of 23 kb was identified to be responsible for the internode diameter and explained 11% of the phenotypic variation. *Lac09g006860* (*LacCRWN3*), encoding a nuclear lamina protein involved in the control of nuclear morphology, was the only gene harbored in *qID2*. Sequence alignment showed completely different promoter sequences between the two parental alleles of *LacCRWN3* except for some nonsynonymous single nucleotide polymorphisms (SNPs) in exons, and the expression level in thick-stem P93075 was distinctively higher than that in thin-stem S1174. According to the natural variation analysis of a population of 183 inbred lines, two main haplotypes were found for *LacCRWN3*: the P93075-like and S1174-like, with the former haplotype lines exhibiting significantly thicker internode diameters than those of the latter haplotype lines. It showed that *LacCRWN3*, as the only CRWN3 gene in Cucurbitaceae, was the most likely candidate gene regulating the internode diameter of *Luffa*. Our findings will be beneficial for deciphering the molecular mechanism of key phenotypic traits and promoting maker-assisted breeding in *Luffa*.

## Introduction

*Luffa* is a monoecious and cross-pollinated diploid belonging to the Cucurbitaceae family (Wu et al., 2016). The *Luffa* genus consists of nine species (Filipowicz and Schaefer, 2014), among which *Luffa acutangula* (Ridge gourd) and *Luffa cylindrica* (Sponge gourd) are domesticated and cultivated worldwide, especially in India, China, Thailand, Central America and Africa (Rabei et al., 2013; Wu et al., 2014). Generally, young *Luffa* fruit commercially produced as an edible vegetable is rich in nutrients, such as proteins, citrulline, vitamins, trace elements and crude fiber (Tyagi et al., 2020). The fruit is also rich in bioactive compounds for medicinal treatment, such as flavonoids, alkaloids, sterols and glycosides (Gowtham et al., 2012; Abdel-Salam et al., 2019). For the mature/old fruit, it includes a tightly arranged network composed of large amounts of fiber, which can be utilized as cleaning products, packaging and industrial materials (Zhang et al., 2007; Papanicolaou et al., 2015). Therefore, *Luffa* should be well-studied as a commercially cultivated crop with nutritional, medicinal, and industrial values.

Several strikingly complementary characteristics have been identified between the two domesticated cultivars: *Luffa cylindrica* has smooth and cylindrical fruit, strong adaptability and disease resistance; while *Luffa acutangular* has ridge gourd fruit, small pale-yellow flowers, good fruit quality and performances during storage and transportation. Furthermore, it is reported that strong heterosis effects exist between the two domesticated cultivars (Wu et al., 2016). However, under natural conditions, the sponge gourd’s flowers usually open between 4:00 and 6:00 a.m., while the ridge gourd bloom between 4:00 and 6:00 p.m. The nearly 12-h gap in the daily flowering time hinders their natural hybridization and gene flow. Thence, in-depth investigation of the genetic and molecular basis of these phenotypic traits in both cultivars is of great significance to promote the utilization of interspecific heterosis and the breeding of new *Luffa* varieties with excellent agronomic performances.

The cucurbit model plant cucumber has been extensively studied to dissect the genetic architecture of important agronomic morphology (Pan et al., 2020a; Pan et al., 2020b; Chen et al., 2021; Shan et al., 2021). In addition to a large number of identified quantitative trait loci (QTLs) (Pan et al., 2020a; Wang et al., 2020), certain genes for corresponding traits, including fruit shape (Jiang et al., 2015; Xin et al., 2019; Zhao et al., 2019; Zhang et al., 2020b), inflorescence architecture (Wen et al., 2021), flowering time (Cai et al., 2020), plant height (Wang et al., 2017a) and stem diameter (Yang et al., 2022), have been characterized, which have considerably facilitated the genetic and molecular breeding of cucumbers. Additionally, a series of QTLs responsible for key phenotypic traits have also been detected in other cucurbits, including melon and watermelon (Ren et al., 2018; Guo et al., 2020; Pan et al., 2020b; Cao et al., 2021).

In comparison, scientists have mainly focused on the traditional *Luffa* breeding program in past decades, and the genetic and molecular research is relatively insufficient. Wu et al. (2016) constructed an interspecific genetic linkage map between *Luffa acutangula* and *Luffa cylindrica* based on 177 expressed sequence tag-derived simple sequence repeat (EST-SSR) markers distributed in 14 linkage groups (LGs), which spanned 1436.12 cM with an average of 102.58 cM per LG, and six putative QTLs associated with reproductive isolation traits were identified. The recent publications on the genomes of *Luffa acutangul*a and *Luffa cylindrica* (Wu et al., 2020; Zhang et al., 2020a; Pootakham et al., 2021) provide useful resources for developing high-throughput genomic variations to improve the efficiency of loci or gene mining. Lou et al. (2020) constructed a high-density genetic map with the genetic background of *Luffa cylindrica*, in which 3701 polymorphic markers based on specific-locus amplified fragment sequencing (SLAF-seq) were mapped to 13 LGs and the map spanned a total distance of 1518.56 cM. Using this genetic map, a LTR Copia-type retrotransposon harbored in the target QTL was predicted as the candidate gene responsible for CMV resistance. Through bulk-segregant analysis sequencing (BSA-Seq) and comparative genomics analyses, a dwarfism gene encoding a GA3ox was mapped using an F_2_ *Luffa acutangular* population, and the large insertion in the mutant allele might account for the defective GAs biosynthesis and dwarf phenotype in WJ209 (Zhao et al., 2021). Moreover, transcriptome and sRNAome, and degradome have also been used for revealing network associated with fruit skin coloration in *Luffa cylindrica* (Sun et al., 2022). To our knowledge, based on the interspecific genetic background between *Luffa acutangula* and *Luffa cylindrica*, no studies have been reported on the comprehensive and high-resolution analyses of the genetic architecture for key agronomic traits.

In the current study, a high-density genetic linkage map across *Luffa* interspecies was constructed using SLAF-seq-based single nucleotide polymorphism (SNP) markers. QTL mapping for important agronomic traits was further performed and key candidate genes were evaluated. In particular, a key candidate gene (*LacCRWN3*) controlling the internode diameter was characterized, and its differential expression profiles associated with the sequence variations in promoter regions might be the main causation for stem thickness divergence between the thin-stem parental line S1174 and thick-stem parental line P93075. Meanwhile, two distinct haplotypes of *LacCRWN3* were found to be responsible for the internode diameter variation in natural population. This study will facilitate the molecular research and maker-assisted breeding in *Luffa*.

## Materials and methods

### Plant materials and growing conditions

The BC_1_ mapping population comprising 110 individuals was derived from a cross between inbred lines P93075 (*Luffa cylindrica*) and S1174 (*Luffa acutangula*), with female line S1174 as the recurrent parent. *Luffa cylindrica* and *Luffa acutangular* are two domesticated *Luffa* cultivars that could be distinguished by different characteristics. The former has smooth and cylindrical fruit, and bright yellow flowers that open early in the morning, whereas the latter has ridged gourd fruit with flowers that bloom late in the afternoon (**Supplementary Figure S1**).

Additionally, a core panel of 183 *Luffa* inbred lines was used for haplotype analysis of the candidate gene. The panel included 94 *Luffa acutangular* and 89 *Luffa cylindrica*, exhibiting abundant genetic and phenotypic diversity.

Field experiments were carried out in Baiyun Field Trial Base of Guangdong Academy of Agricultural Sciences (23° 15′ N, 113° 27′ E) during the spring-summer season of the year 2015 for the BC_1_ population, and the field management followed local practices. The parental and offspring lines were grown in a completely randomized design across eight rows of 20 individuals. Each row was 8 m long with a row spacing of 0.4 m. For the 183 inbred lines, the field trials were conducted in the same location in the summer-autumn of 2021 and spring-summer of 2022. Each inbred line was grown in two rows, with 3 m long and 0.7 m apart, and 12 individuals were planted for each line.

### Phenotyping and phenotypic data analysis

During the growing season of *Luffa*, 110 BC_1_ individuals and their parental lines were investigated for four categories of agronomic variables: maturity-related traits, flower-size-related traits, plant-growth-related traits and fruit-related traits. At the flowering stage, the first node for the male flower (FMFN), female flower (FFFN) and fruit (FFRN), daily flowering time (FT) and male flower diameter (MFD) were measured to evaluate plant maturity and flower size. During the late stage of plant growth, the main stem and branch-related traits were determined, including the total number of internodes (TNI), length and diameter of the 7th, 8th and 9th internodes (IL_7th, IL_8th, IL_9th, IL_AV, ID_7th, ID_8th, ID_9th, ID_AV), growth rate (GR), branch number (BN) and total branch length (TBL). During the fruit development stage, age-related parameters such as fruit length and diameter (YFL, OFL, YFD, OFD), as well as fruit peduncle length and diameter (YFPL, OFPL, YFPD, OFPD), were measured. For the 183 inbred lines, only the internode diameter was examined. A description of the phenotypic traits is presented in **Supplementary Table S1.**

All phenotypic statistical analyses were performed in R (v3.4.2). The Pearson correlation coefficients among different traits were computed and visualized by the Corrplot and Hmisc packages. The Skewness and Kurtosis functions in the fBasics package were used for the normal test. Phenotypic frequency distributions were plotted using OriginPro 9.0.0.

### Genomic DNA extraction

The genomic DNA was extracted from three-week-old leaves of all samples, including the two parental lines and their 110 BC_1_ progeny individuals, as well as the 183 inbred lines. DNA extraction was performed using the modified cetyltrimethylammonium bromide (CTAB) method, and then purification with a plant genomic DNA extract kit (Tiangen Inc., Beijing, China). DNA concentration and quality were measured by NanoDrop ND-1000 Spectrophotometer and 1% (w/v) agarose gel electrophoresis.

### SLAF-seq and genotyping

The strategy of SLAF-seq described by Sun et al. (2013) was utilized for the BC_1_ population and its parental lines with some modifications. Briefly, the cucumber(http://www.icugi.org/cgi-bin/ICuGI/genome/home.cgi?organism=cucumber&ver=2)

was selected as the reference genome for predicting enzyme digestion due to a lack of the *Luffa* genome in 2016, and the restriction enzymes of HaeIII+Hpy166II were specified to digest the genomic DNA. Afterwards, the fragments ranging from 314 to 364 bp were purified to construct the SLAF library and perform pair-end sequencing (Each end 125 bp) on an Illumina HiSeq 2500 platform (Illumina, Inc., San Diego, CA, USA) by Biomarker Technologies Corporation (Beijing, China). A control experiment with *Oryza sativa ssp. japonica* was conducted to evaluate the accuracy and reliability of the SLAF library construction and sequencing. The results showed that the enzyme digestion efficiency of control data was 91.76%, and the paired-end mapping efficiency was 78.56%, indicating that the experimental process was effective. After trimming adapters and filtering low quality raw reads of SLAF (Reads with > 10% unidentified nucleotides [N]; > 50% bases having Phred quality score ≤ 5), the clean reads were mapped to reference genome *Luffa acutangular* S1174 (Unpublished data) using Burrows Wheeler Alignment (BWA, v0.7.8) (Li and Durbin, 2009). For the uniquely mapped reads, SNP calling was performed with Genome Analysis Toolkit (GATK, v3.2-2) (Mckenna et al., 2010), and the detected SNPs were designated as the final variations for subsequent analysis.

To develop available molecular markers for construction genetic linkage map, we first identified SNPs that were bi-allelic, homozygous and polymorphic between parental lines, and a set of targeted SNPs of aa × bb type was identified. Furthermore, the genotypes of 110 BC_1_ individuals at these polymorphic loci were extracted and filtered according to the following criteria: SNPs with multiple alleles that were not inherited from parents were removed; SNPs genotyped in more than 99% of offspring individuals were retained; SNPs with significant segregation distortion (*χ* ^2^ test, *P* < 0.001, D.F. = 2) were excluded. Finally, a collection of high-quality SNP markers was obtained for linkage map construction.

### Genetic linkage map construction and QTL mapping

The *Luffa* genetic map was constructed by the Lep-MAP3 software, with the maximum likelihood method employed to order the markers within each LG, and Kosambi mapping function used for converting recombination percentages to genetic distances in cM (Rastas et al., 2017).

QTL analysis was performed using the composite interval mapping (CIM) model by WinQTL Cartographer software (v2.5) (Wang et al., 2012). The logarithm of odds (LOD) threshold for QTL detection was determined based on 1000 permutations at a significance level of *P* < 0.05 for every trait with the function of permutation test in MapQTL (v6.0) (Van Ooijen, 2009). At the highest probability peak, the phenotypic variance explained by each QTL was achieved.

### Candidate genes identification and gene ontology analysis

All the genes within the QTLs were identified as potential candidate genes. The most likely genes were further selected according to their functional annotation and orthologous gene prediction in other species. For the candidate genes of a certain trait, gene ontology (GO) enrichment analysis was performed using Singular Enrichment Analysis tool in agriGO1.2 (Tian et al., 2017). A false discovery rate of 0.05 was used to identify significant GO terms.

### Haplotype analysis of the candidate gene

Whole genome re-sequencing of the 183 core *Luffa* lines was performed to examine the haplotype distribution of the candidate gene. The following is a summary of the re-sequencing and SNP detection pipeline: sequencing was done on the DNBSEQ platform (China National GeneBank, Shenzhen, China) by 150 bp paired-end sequencing with an average depth of approximately 6.48 ×. Adapters were trimmed and low quality reads (Reads with > 10% unidentified nucleotides [N]; > 50% bases having Phred quality score ≤ 5) were filtered before mapping the clean reads to the S1174 reference genome (Unpublished data) using BWA (v0.7.12) (Li and Durbin, 2009). SNP and insertion and deletion (InDel) calling were performed with GATK (v4.2.2.0) (Mckenna et al., 2010), and ANNOVAR was used for functional annotation (Wang et al., 2010). The obtained variations were further filtered with a missing rate < 0.5 and a minor allele frequency > 0.05. Subsequently, we extracted the SNPs and InDels around the candidate gene in each *Luffa* accession to examine the haplotypes.

### Phylogenetic tree construction

Through sequence alignment, two CRWN homologs (CRWN3 and CRWN4) were found in *Luffa*, and the amino acid sequences similar to both proteins were downloaded from public databases of maize (*Zea mays*), rice (*Oryza sativa*), *Arabidopsis thaliana* and other eight Cucurbitaceae crops. The sequences were aligned by ClustalW, and a maximum-likelihood phylogenetic tree was constructed with 1000 bootstrap replicates using MEGA (v7.0.26). Online software MEME was used to predict the protein motifs with a maximum of 10 motifs and a maximum width of 100.

### RNA extraction and quantitative real-time PCR

Total RNA was extracted from the 7th, 8th and 9th internode of main stem in three biological replications for each parent, with a quick RNA isolation kit (TransGen Biotech, China), and complementary DNA (cDNA) was synthesized by the Transcript One-Step gDNA Removal and cDNA Synthesis SuperMix (TransGen Biotech, China) using a 20 μl reaction system. The quantitative real-time PCR (qRT-PCR) was performed using TB Green Premix Ex Taq II (Takara, Japan) in three replications for each sample. *Luffa 18S rRNA* gene was used as an internal control and relative expression was calculate by 2^-ΔΔct^ method (Livak and Schmittgen, 2001).

Primers used for qRT-PCR were listed as below: the *18S rRNA* gene (F: 5’-GTGTTCTTCGGAATGACTGG-3’, R: 5’-ATCGTTTACGGCATGGACTA-3’); the candidate gene *Lac09g006860* in S1174 (F: 5’-CGTAATGGAGCGCAAAGATCGG-3’, R: 5’-CTTGCCCTAGTTGGTCATACTT-3’) and P93075 (F: 5’- CGTAATGGAGCGCAAAGATCGG -3’, R: 5’-TCTTGCCCTAGTTGGTCGTACTT -3’).

### Statistical analysis

One-way ANOVA followed by Duncan’s or Dunnett’s T3 multiple range tests (*P* < 0.05) or two-tailed student’s *t* test (*P* < 0.05) were performed to evaluate the data differences by SPSS 18.0.

## Results

### Analysis of high-throughput sequencing data and SNP markers development

After SLAF-seq and raw reads filtering, a total of 2.33 G, 2.11 G and 39.75 G clean data were obtained for the maternal and paternal parents and BC_1_ population. Each offspring individual’s average data was greater than 0.36 G, with the average Q30 of 91.59% and guanine cytosine (GC) content of 40.68% (**Supplementary Table S2**). The average mapping rate of all progeny samples was 96.46%, and their average sequencing depth was 1.45 ×, ranging from 1.24 × to 1.71 × (**Supplementary Table S2**). The results indicated that the sequencing quality was high and the data could be used for subsequent analysis.

Through statistical analysis of SNP data between paternal and maternal parents, 388,402 polymorphic SNPs following the genetics’ two allelic general encoding rules were developed altogether, and 319,601 fell into the aa × bb segregation pattern (**Supplementary Table S3**). The genotypes of 110 BC_1_ individuals were identified at these targeted polymorphic loci and further filtered with criteria described in method section. Ultimately, a collection of 4,798 polymorphic SNP markers was obtained and used for linkage map construction.

### Construction of high-resolution linkage map for *Luffa*

**Figure 1:**
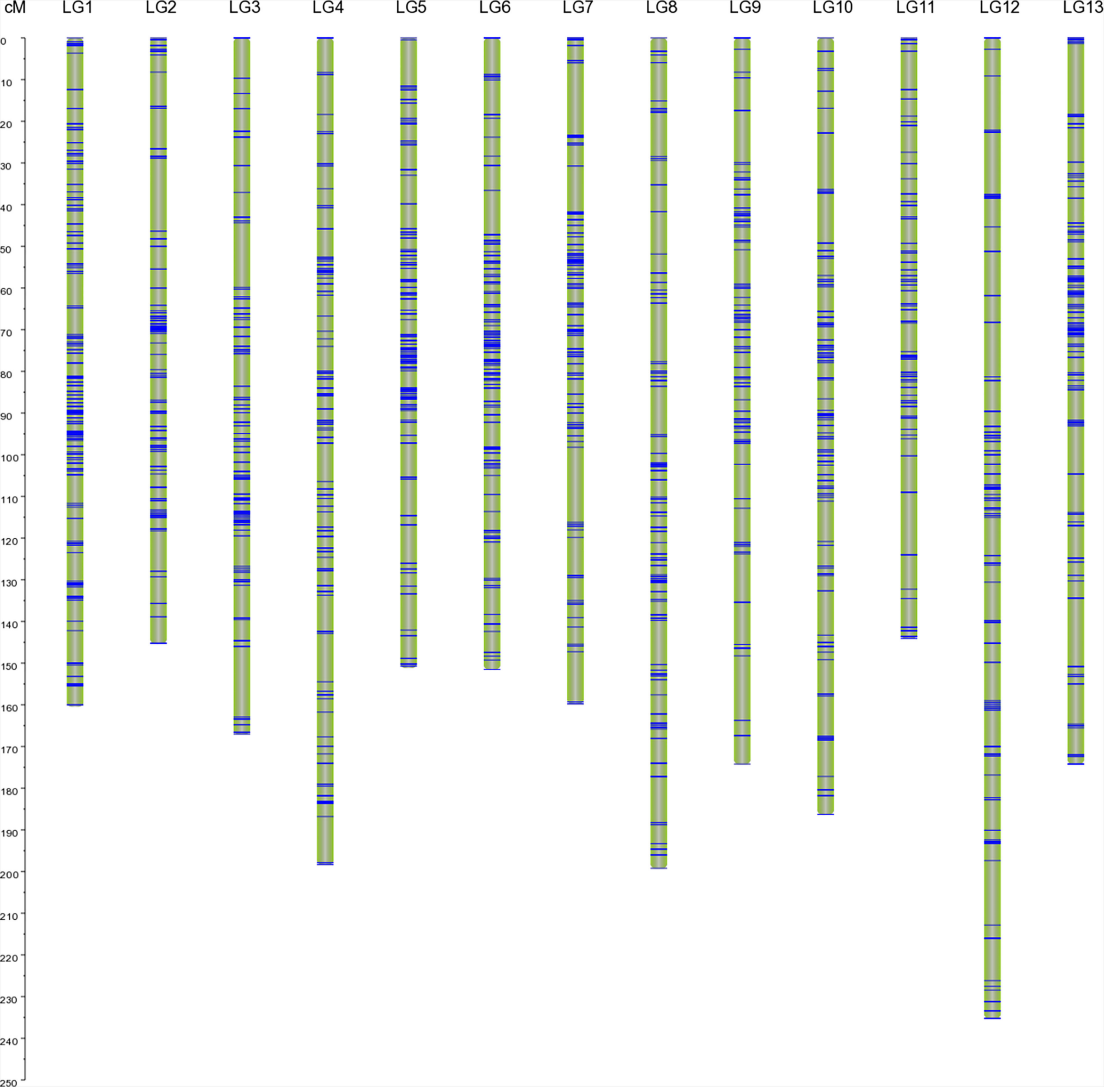
An interspecific high-density genetic linkage map of *Luffa* with 13 linkage groups. Single nucleotide polymorphism (SNP) markers are highlighted with blue lines on each linkage group (LG).

By using the Lep-MAP3 software, 4,673 markers were successfully mapped onto the linkage map of BC_1_ population, resulting in an effective mapping ratio of available SNPs approximately 97.58%. The genetic map included 13 LGs consistent with the haploid chromosome number of *Luffa*, spanning a total of 2246.74 cM with an average distance of 0.48 cM between adjacent markers (**Figure 1**; **Supplementary Table S4**). The genetic length within LGs varied from 144.09 cM in LG11 to 235.25 cM in LG12, with an average of 172.83 cM, and the number of markers ranged from 237 in LG11 to 481 in LG1, with an average of 359.46 (**Supplementary Table S4**). LG1 was the densest group, with a total distance and an average marker interval of 160.45 cM and 0.33 cM, respectively (**Supplementary Table S4**). The number of markers with a gap less than 5 cM accounted for 97.15% of the linkage map, indicating relatively good distribution uniformity. Since the genome size of *Luffa acutangular* S1174 has been estimated to be 776.49 Mb (Unpublished data), the average recombination rate across all LGs was approximately 2.89 cM/Mb. This is the highest density genetic map of *Luffa* reported to date (**Figure 1**), which is generally considered suitable for QTL mapping.

### Phenotypic variation analysis

Significant variations were observed for all phenotypes in the two parental lines (**Table 1**). P93075 showed higher first node for male flower (FMFN), female flower (FFFN) and fruit (FFRN), and earlier diurnal flowering time (FT) than those of S1174, (**Table 1**; **Supplementary Figures S2**, **S3**), indicating that S1174 was an early-maturing variety in terms of overall plant growth and development. For the plant-growth related traits, S1174 exhibited lower growth rate (GR), total number of internodes (TNI), branch number (BN), total branch length (TBL), and internode diameter (ID) (**Table 1**; **Supplementary Figures S2**, **S3**). The results suggested that the whole plant growth momentum of P93075 was more vigorous than that of S1174, whereas the latter might perform bettered in the 7th, 8th and 9th internode lengths (ILs). The fruit length of S1174 was longer compared to that of P93075 during the whole fruit development period, and its fruit diameter was larger at the young fruit stage but thinner at the old fruit stage (**Table 1**; **Supplementary Figures S2**, **S3**). Meanwhile, distinct phenotypic variations in the derived BC_1_ population were also investigated, with the coefficient of variation (*CV*) ranging from 5.52 in FT to 82.14 in TBL (**Table 1**).

**Table 1:** Basic description of phenotypic data in parental lines and the BC1 population.

Category	Traits	Parents		BC1 lines					
		S1174	93075	Min	Max	Mean ± SE	Skewness	Kurtosis	*CV*
Maturity related traits	FMFN	2.50	16.56	1.00	12.00	4.10 ± 0.20	1.45	1.98	51.07
	FFFN	6.00	33.00	4.00	65.00	20.74 ± 1.18	1.26	1.97	59.51
	FFRN	9.23	34.50	7.00	66.00	26.85 ± 1.21	0.90	0.63	47.15
	FT	17.17	5.00	17.87	22.62	19.72 ± 0.10	1.01	0.83	5.52
Flower size related traits	MFD (mm)	8.63	14.79	5.60	12.01	9.00 ± 0.12	0.18	-0.03	14.01
Plant growth related traits	GR	0.89	0.90	0.50	2.17	1.30 ± 0.03	0.07	0.14	24.94
	TNI	27.33	29.50	21.00	44.00	32.28 ± 0.42	-0.29	-0.09	13.51
	IL_7th (cm)	12.92	8.91	2.50	19.30	10.97 ± 0.33	-0.19	-0.16	31.43
	IL_8th (cm)	12.46	8.94	3.00	20.30	11.40 ± 0.36	0.14	-0.38	33.16
	IL_9th (cm)	13.02	9.77	3.00	20.80	11.78 ± 0.33	0.15	-0.06	29.23
	IL_AV (cm)	12.80	9.21	5.07	18.17	11.38 ± 0.29	0.10	-0.58	26.72
	ID_7th (mm)	4.55	4.75	3.00	10.50	5.57 ± 0.09	1.12	5.33	17.34
	ID_8th (mm)	4.77	4.82	3.00	10.30	5.47 ± 0.09	1.11	5.14	17.55
	ID_9th (mm)	4.67	4.91	3.40	9.80	5.37 ± 0.08	1.08	6.22	15.47
	ID_AV (mm)	4.66	4.83	3.13	8.07	5.47 ± 0.07	0.08	0.91	14.29
	BN	1.85	5.10	1.00	13.00	4.55 ± 0.28	0.80	0.06	63.53
	TBL (cm)	103.30	171.05	0.00	568.00	159.67 ± 12.51	0.92	0.34	82.14
Fruit related traits	YFL (cm)	41.00	31.20	11.80	64.50	42.65 ± 0.86	-0.24	0.58	21.04
	YFD (mm)	64.02	63.80	33.00	76.70	60.07 ± 0.73	-0.35	0.88	12.80
	YFPL (cm)	12.00	14.07	6.80	47.40	12.98 ± 0.42	4.63	35.56	33.59
	YFPD (mm)	9.20	10.34	7.40	15.40	11.13 ± 0.15	0.13	-0.22	14.39
	OFL (cm)	47.38	40.60	29.80	82.70	48.59 ± 0.87	0.71	1.23	18.86
	OFD (mm)	63.28	98.28	34.80	110.00	75.70 ± 1.22	-0.58	1.14	16.96
	OFPL (cm)	11.13	13.70	6.50	21.80	12.59 ± 0.27	0.85	1.34	22.49
	OFPD (mm)	11.95	11.44	3.20	16.10	11.40 ± 0.22	-0.91	1.16	19.94

The skewness and kurtosis test demonstrated that all the traits were approximately normally distributed, highlighting that they followed the genetic characteristics of a quantitative trait and might be controlled by multiple genes (**Table 1**; **Supplementary Figures S2**, **S3**). Pearson correlation analysis showed that there were significant correlations among many traits within one category, and the closest correlations were found among IL and ID (0.83-0.90, *P* < 0.001), followed by that between FFFN and FFRN (0.83, *P* < 0.001) (**Supplementary Figures S4A, C**). As expected, both length and diameter between fruit and fruit peduncle were positively correlated, and TNI was also found to be positively correlated with BN and TBL (**Supplementary Figures S4B, C**). While for traits across different categories, the strongest correlations were found between FFFN, FFRN and TNI with the *r* of 0.34 (*P* < 0.01) (**Supplementary Table S5**).

### QTL mapping

**Figure 2:**
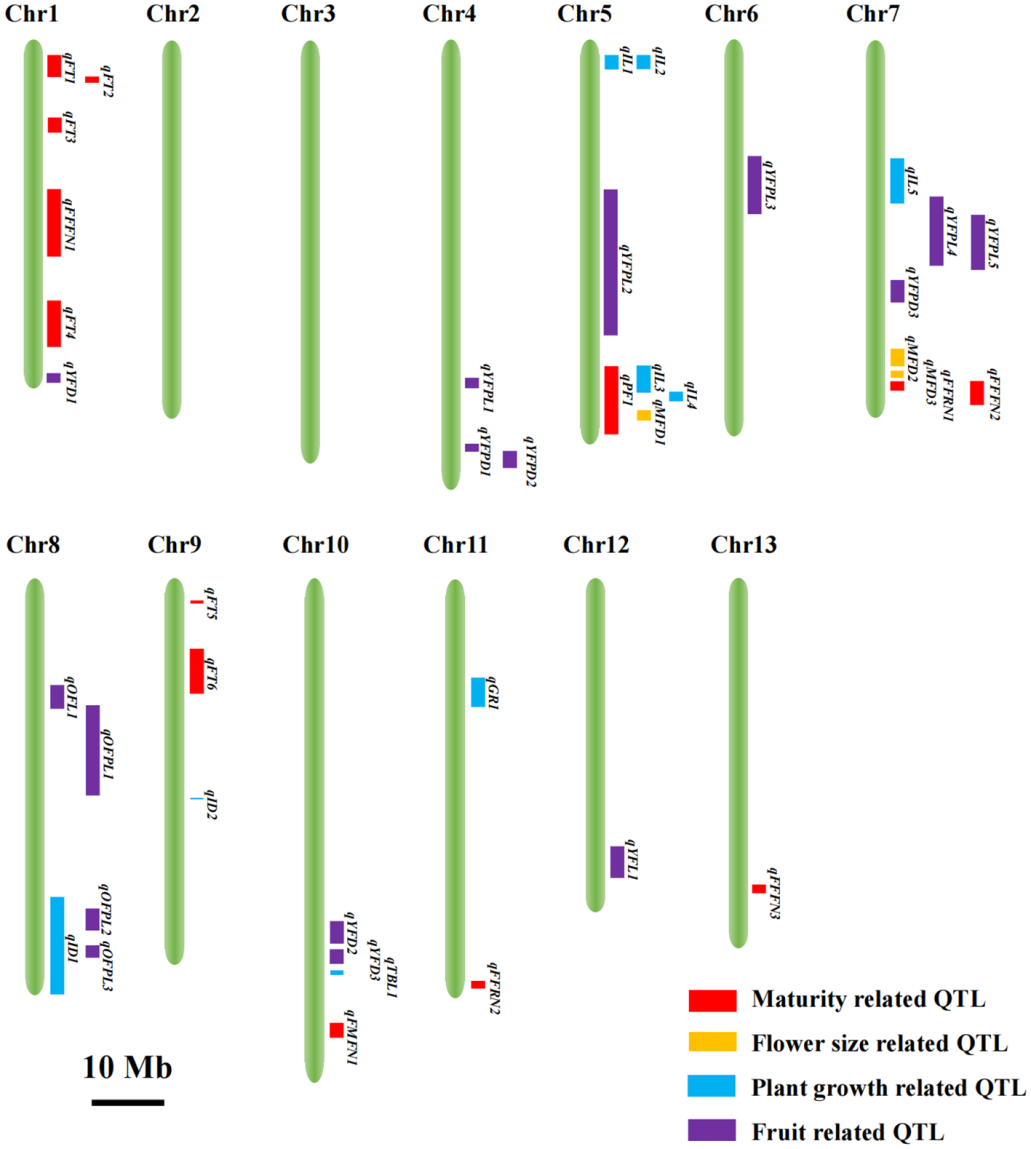
Visualization of the chromosomal locations of the 40 QTLs. The red, yellow, blue and purple rectangles represent the QTLs related to maturity, flower size, plant growth and fruit, respectively. The 10 Mb bar indicates the size of regions in the reference genome *Luffa acutangular* S1174. Chr, chromosome.

Totally, 40 QTLs associated with 17 traits were distributed across 11 chromosomes of *Luffa* except chromosomes 2 and 3, and the LOD scores ranged from 3.46 to 23.12, while no QTL was detected for eight traits including TNI, BN, IL_AV, ID_7th, ID_8th, ID_9th, old fruit diameter (OFD) and old fruit peduncle diameter (OFPD) (**Figure 2**; **Supplementary Table S6**). The estimated phenotypic variation explained (PVE) by the significant locus individually varied from 5% to 47%, with 80% of them accounting for > 10% and 15 loci > 15%, suggesting that they were the major effect QTLs responsible for the genetic controls of these traits. Among the 40 QTLs, the additive effects of 10 QTLs were derived from S1174 alleles, whereas those of the other 30 QTLs were from P93075 alleles.

For maturity-related traits, 12 QTLs were mapped to chromosomes 1, 7, 9, 10, 11 and 13 with PVE values of 5-26% (**Figure 2**; **Supplementary Table S6**). QTL *qFT5* had the largest effect, which explained a phenotypic variation of 26%, and the allele from P93075 was correlated with 1.17 h advanced in daily flowering time. The followed largest effect QTLs, *qFFRN1* and *qFFFN1* with PVEs of 18% and 17.2%, were located on chromosomes 7 and 1, and the allele from S1174 at the corresponding locus was responsible for a decrease in FFRN and FFFN of 11.41 and 10.58, respectively. *qFFRN2*, another large-effect QTL with ≥ 15% PVE, located on chromosome 11, and the S1174 allele contributed to an increase of 9.8 in FFRN.

Meanwhile, three QTLs on chromosomes 5 and 7 were identified for male flower dimeter, accounting for 40% of the total phenotypic variance, and those alleles with increasing effect were all from P93075 (**Figure 2**; **Supplementary Table S6**).

For plant-growth-related traits, nine QTLs were found localized on chromosomes 5, 7, 8, 9, 10 and 11, with PVEs ranging from 10% to 23%, of which the increasing effects for *qGR1*, *qIL3*, *qIL4*, *qID2* and *qTBL1* were from P93075, and those for other QTLs of *qID1*, *qIL1*, *qIL2* and *qIL5* from S1174 (**Figure 2**; **Supplementary Table S6**).

For fruit-related traits, 16 QTLs were detected across eight chromosomes, contributing to PVE values of 8%-47% (**Figure 2**; **Supplementary Table S6**). Among them, *qYFPL5* and *qYFPL2*, which are located on chromosomes 7 and 5, had the greatest effects and accounted for 47% and 40% of variance in young fruit peduncle length (OFPL). The P93075 allele at *qYFPL5* and S1174 allele at *qYFPL2* had the increased effects of YFPL. Regarding to old fruit peduncle length, two QTLs, *qOFPL2* and *qOFPL3* on chromosome 8, were detected, explaining 24% and 19% of total phenotypic variation, and the P93075 alleles at these two loci increased the OFPL by 2.79 cm and 2.52 cm, respectively. The three QTLs related to young fruit, *qYFD1* on chromosome 1, *qYFD2* on chromosome 10 and *qYFL1* on chromosome 5, accounted for ≥ 15% PVE, and the alleles from S1174, P93075 and P93075 were responsible for the increase in the corresponding traits of 6.29 mm, 7.32 mm and 7.34 cm, respectively.

### Candidate genes analysis for the three key types of agronomic phenotypes

The genes underlying the six QTLs for daily flowering time, five QTLs for fruit shape and seven QTLs for internode development were retrieved, and a total of 2,980 genes were detected.

The six QTLs detected for daily flowering time were found to harbor 633 genes. Based on GO annotation and orthologous gene prediction, four key genes were prioritized to be the most likely genes (**Supplementary Table S7**). *Lac01g003380* and *Lac01g013890* were two genes enrichened in the circadian rhythm network for modulating flowering time *via* the photoperiod pathway, and they encoded a phytochrome C and a casein kinase II subunit beta (CK2β), respectively. Phytochrome C was reported as the photoreceptor to transmit signals to the circadian clock (Kircher et al., 2002), and CK2 acted as a ubiquitous Ser/Thr kinase to phosphorylate the central clock components CCA1 and LHY (Mulekar and Huq, 2012). The two *Arabidopsis* orthologs, *Lac01g012970* (*AtSEP3*) and *Lac01g012980* (*AtSOC1*), both encoded MADS-box transcription factors, were also involved in the regulation of flowering time and flower morphogenesis.

A total of 731 genes were found underlying the five QTLs regions detected for fruit shape, and putative six genes were prioritized, three of which were enriched in cell cycle, two in plant hormone responses, and one selected for its significance in other crops (**Supplementary Table S7**). Two cyclins (*Lac10g017950* and *Lac10g018520*) and one CDK inhibitor gene (*Lac10g016980*) were shown to modulate the cell division and expansion of fruit by changing their expression patterns in cell cycle progression (Anwar et al., 2019). *Lac10g015340* and *Lac10g015350*, both encoding auxin-responsive protein IAA14, were predicted to play roles in cell enlargement during fruit development. *Lac10g015060* (*SUN*, *Cla011257*) encoding an IQ-DOMAIN14 protein was another most likely candidate gene regulating fruit shape.

For the five QTLs of internode length, 551 genes were detected and 11 were prioritized according to previous findings that the improved plant height of high-yielding varieties generated during the ‘green revolution’ was primarily due to the significant change of GA pathway, and its crosstalk with other phytohormones (Wang et al., 2017b). These candidate genes included *GA20ox* and *gibberellin receptor GID1B-like* from GA biosynthesis and signaling; *TIR1*, *auxin-induced protein AUX28-like* and *22D-like* from auxin metabolism; five ethylene-responsive transcription factors in ethylene signaling; and *TIFY 9-like isoform X2* from JA pathway (**Supplementary Table S7**).

In addition, Two QTLs for the internode diameter, *qID1* and *qID2*, were detected. We mainly focused on *qID2*, which was located on chromosome 9 with a minimum distance of 23 kb and explained 11% of the phenotypic variation (**Figure 2**; **Supplementary Table S6**). Fascinatingly, *qID2* harbored only one candidate gene, *Lac09g006860*, which encodes a CROWDED NUCLEI 3 (LacCRWN3) protein involved in the regulation of nuclear structure.

### *LacCRWN3* responsible for the internode diameter of main stem in *Luffa*

**Figure 3:**
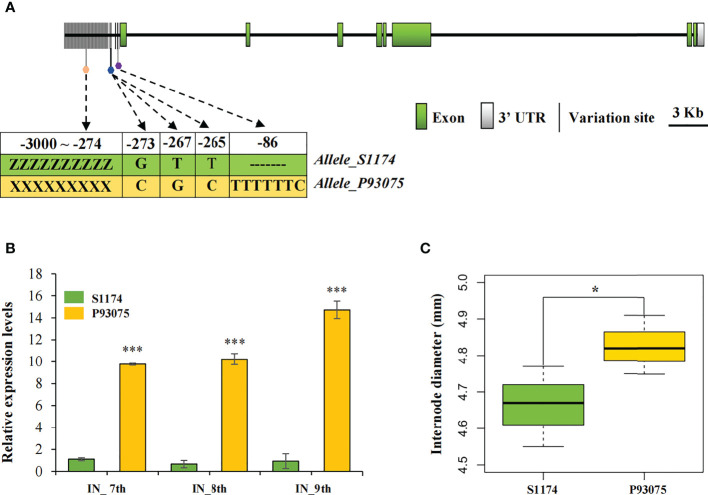
Candidate gene *LacCRWN3* targeted by *qID2* responsible for internode diameter. **(A)** Schematic representation of the structure and sequence variations of *LacCRWN3*. The white box represents the 3’ un-translated region (UTR), and the green boxes represent exons. The upstream region before the first exon indicates the 5’ UTR and promoter region. The vertical black lines indicate variations between S1174 and P93075 in the upstream region. The pentagons represent variation sites in the major cis-elements, different colors indicate different cis-elements. ZZZZZZZZZ and XXXXXXXXX refer to completely different allelic sequences in the region -3000 to -274 upstream of the start codon ATG. Variations of the marked sites between alleles from S1174 and P93075 are displayed in green and yellow backgrounds, respectively. **(B)** Expression profiles of *LacCRWN3* in the 7th, 8th, and 9th internode of parental lines S1174 and P93075. **(C)** Phenotypic variation of internode diameter between parental lines S1174 and P93075. Results indicate mean ± SE of three replicates, ***P < 0.001; *P < 0.05.

*Lac09g006860* was an extremely large gene of 31,127 bp and its paralogue in P93075 was *Lcy09g015130* with a length of 53,482 bp, and their amino acid identity reached 98.07%. To detect genomic variations of this candidate gene between parental lines S1174 and P93075, DNA sequences containing the whole genomic regions and the 2000 bp upstream regions of ATG were extracted from the two cultivars’ *de novo* data (Wu et al., 2020; Unpublished data). In the genomic region, 40 SNPs were present in exons, which caused 23 nonsynonymous mutations (Dittmer et al., 2007; Zhao et al., 2016). We further compared the upstream regulatory regions, and a total of six variations including four SNPs and two InDels were detected in 5’ un-translated regions (UTRs), four of which were related to major cis-elements (E-box: 5′-CANNTG-3′; ARE motif) (**Figure 3A**). Compared with P93075, there were three base mutations around the MYC binding motif E-box (265-273 bp upstream of ATG) in S1174, and a 7 bp deletion (-86 bp) was also detected next to the ARE cis-acting regulatory element essential for the anaerobic induction. Interestingly, two alleles of the candidate gene had completely different promoter sequences upstream of 5’ UTR, and many vital regulatory elements related to hormones and abiotic stress were identified. In particular, the unique ABRE motifs related to abscisic acid (ABA) responses were identified in P93075.

Considering that the sequences of this candidate gene *LacCRWN3* were distinctively different in the upstream regulatory regions, we hypothesized that the observed internode diameter variation might be primarily due to the differential gene expression patterns in parental lines. To confirm this speculation, qRT-PCR assays were initially conducted in the 7th, 8th and 9th internode between S1174 and P93075, and the data showed that expression levels of *LacCRWN3* in P93075 were significantly higher than those in S1174 for all the three internodes (**Figure 3B**). Additionally, in P93075, *LacCRWN3* was strongly upregulated from the 7th to 9th internode, while in S1174, the expression levels were weak and maintained almost at the same level for all the three internodes (**Figure 3B**). The results indicated that differential gene expression profiles were tightly linked to the phenotypic variation of the two parental lines, and *LacCRWN3* may act as a positive regulator of internode diameter (**Figures 3B, C**).

### Two haplotypes of *LacCRWN3* identified among the inbred line population

**Figure 4:**
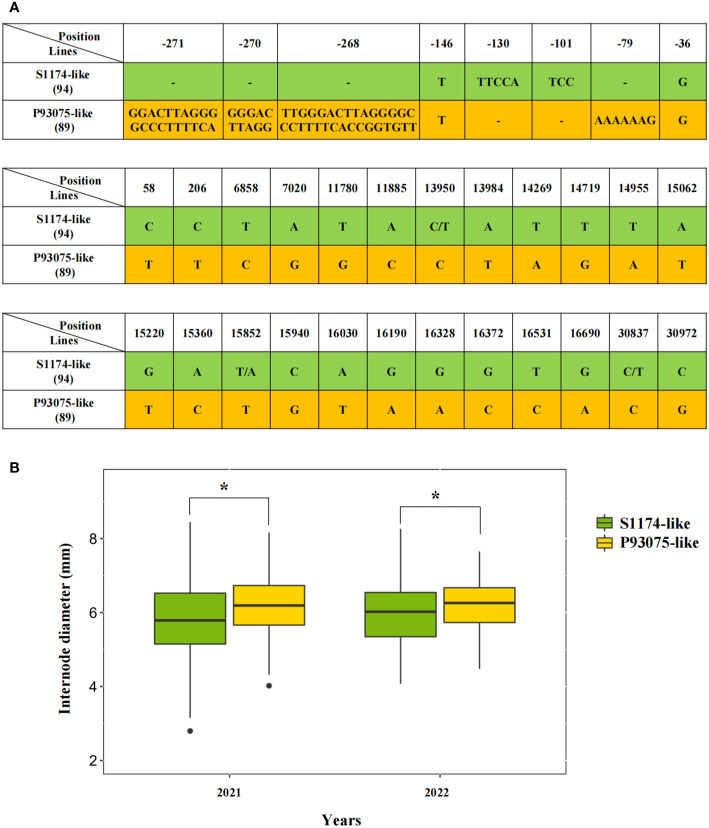
Haplotype analysis of *LacCRWN3* among the 183 core inbred line population. **(A)** Distribution of the nonsynonymous mutation single nucleotide polymorphisms (SNPs) in exons and variations in 5’ un-translated region (UTR) for the two haplotypes of S1174-like and P93075-like. The figures in parentheses denote the number of inbred lines belonging to the corresponding haplotypes. The two haplotypes are displayed in green and yellow backgrounds, respectively. **(B)** The average internode diameter differences between the two haplotypes in the 2021 and 2022 seasons. Results indicate mean ± SE of three replicates, *P < 0.05.

To further explore the functions of vital alleles of *LacCRWN3*, we genotyped natural allelic variations in 183 *Luffa* inbred lines by re-sequencing. Intriguingly, according to the SNPs and InDels covering the entire gene and 2000 bp upstream region of ATG, two main haplotypes of *LacCRWN3* were identified in the population, namely the S1174-like and P93075-like (**Figure 4A**). In total, 50 SNPs were detected in exon regions, among which 24 were nonsynonymous, and eight variations (two SNPs and six InDels) and completely different sequences were found in the 5’UTR and promoter regions, respectively. Statistically, lines with the P93075-like haplotype showed significantly (*P* < 0.5) thicker main stem diameters than those with the S1174-like haplotype (**Figure 4B**), further suggesting that the *P93075-like* allele can thicken the stem of *Luffa*. The results demonstrated that *LacCRWN3* was indeed a key candidate regulatory gene responsible for the variation of internode diameter in *Luffa*.

### *LacCRWN3* being the only *CRWN3* gene in Cucurbitaceae crops

**Figure 5:**
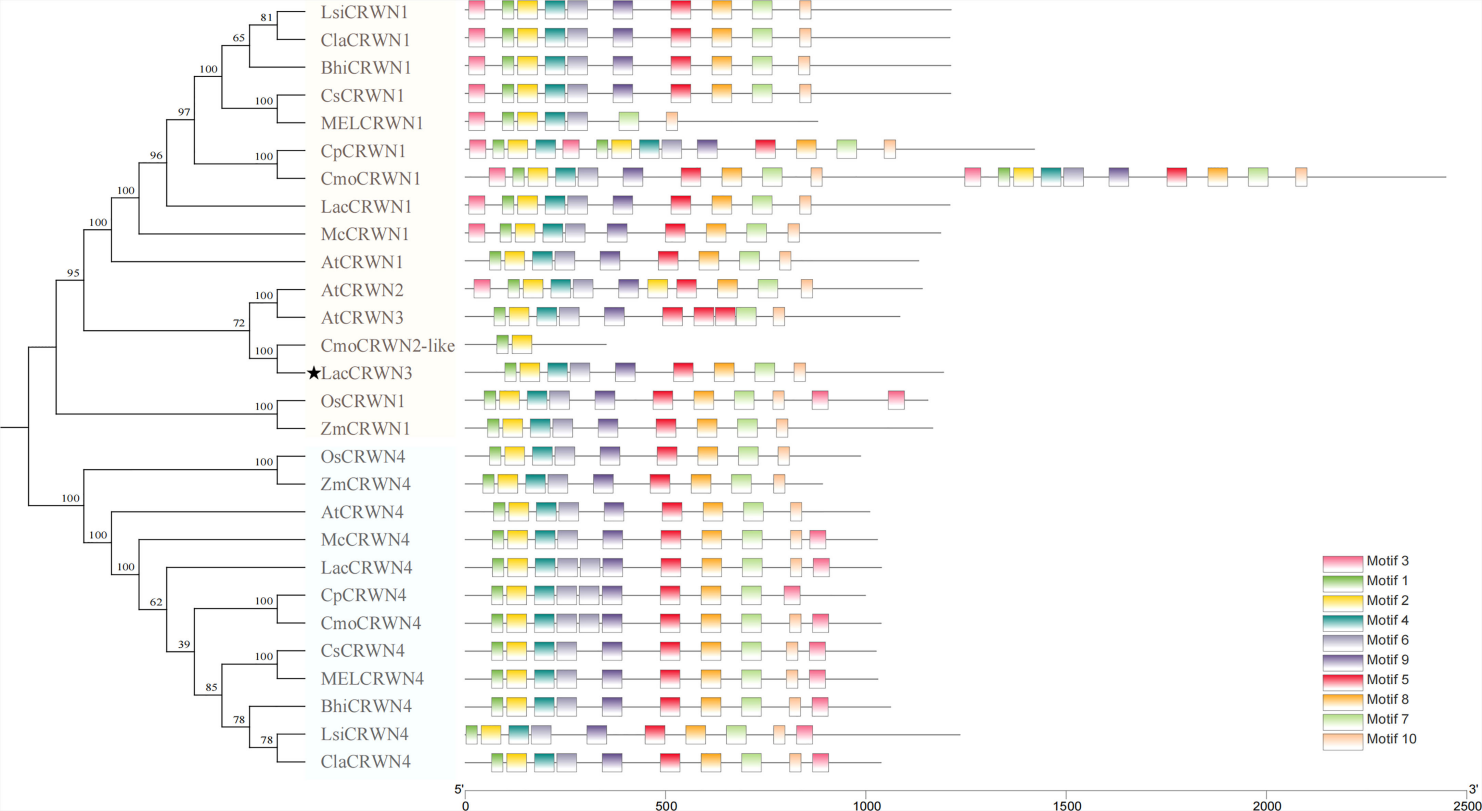
Phylogenetic and conserved motif analysis of *LacCRWN3*. The protein sequences of the LacCRWN3 family were from *Luffa*, the other eight Cucurbitaceae species, *Arabidopsis*, rice and maize.

It has been reported that the CRWN family consists of four members with each contains a large central coiled-coil domain (Dittmer et al., 2007; Wang et al., 2019). To investigate the potential diversification of CRWN family in Cucurbitaceae, the phylogenetic analysis was done (**Figure 5**). The results illustrated that there were three members of the CRWN in *Luffa*, including the CRWN1-like, CRWN3-like (LacCRWN3) and CRWN4-like, while other Cucurbitaceae species possessed only the CRWN1-like and CRWN4-like proteins. Therefore, our targeted LacCRWN3 was the only CRWN3 protein in Cucurbitaceae, which hinted that a special pathway mediated by CRWN3 to regulate the stem development may exist in *Luff* compared with the mechanisms in other Cucurbitaceae.

## Discussion

*Luffa* is an important cucurbit vegetable crop grown worldwide. Strong heterosis effects and strikingly complementary characteristics were found between its two domesticated cultivars, *Luffa acutangular* and *Luffa cylindrica*. To explore the genetic basis underlying important agronomic traits of the two cultivars, we generated an interspecies BC_1_ population of 110 individuals to construct the high-quality genetic map. Simultaneously, QTL mapping and candidate gene identification of important agronomic traits were carried out.

### The most comprehensive dataset of phenotypic analysis in *Luffa*

The two parents of the BC_1_ population, S1174 and P93075, are representative lines of the two commonly cultivated *Luffa* species, *Luffa acutangular* and *Luffa cylindrica*, which could be distinguished mainly by fruit ridge and several other phenotypic characteristics (**Supplementary Figure S1**) (Pootakham et al., 2021). In present study, we measured 25 traits related to maturity, flower size, plant growth and fruit (**Table 1**; **Supplementary Figures S2**, **S3**). The results illustrated distinctive phenotypic variations within parents and the derived BC_1_ population, and S1174 was found to be an early-maturing but late-daily-flowering cultivar with smaller light-yellow male flower, and longer internode length and fruit, while P93075 performed better in the entire plant growth momentum, fruit peduncle length and old fruit diameter. All of these traits exhibited relatively normal distributions in the BC_1_ population, indicating that they are quantitative traits controlling by multiple genes, and the phenotypic distribution pattern of diurnal flowering time was consistent with our previous research of the F_2_ population (Wu et al., 2016).

As expected, significant correlations were discovered among many traits within one category, such as that between FFFN and FFRN (*r* = 0.83, *P* < 0.01). While for traits across different categories, the correlation coefficients were relatively low, with the strongest correlation between FFFN, FFRN and TNI (*r* = 0.34, *P* < 0.01) (**Supplementary Figure S4**; **Supplementary Table S5**). To our knowledge, this is the largest and most comprehensive dataset of phenotypic analysis in *Luffa* across both cultivars of *Luffa cylindrica* and *Luffa acutangula*.

### The first high-density genetic map across interspecies of *Luffa*

In our previous study, a genetic map of *Luffa* using 177 EST-SSR markers was constructed (Wu et al., 2016). However, the genome coverage and resolution of the map were relatively poor because of the low polymorphism of SSR makers. With the development of high-throughput sequencing and bioinformatics technology, several *Luffa* genomes have been published successively in recent years (Zhang et al., 2020a; Pootakham et al., 2021). and our team has completed the *de novo* assemblies of S1174 (Unpublished data) and P93075 (Wu et al., 2020). As a result, high-throughput markers may be developed to efficiently improve the genetic map. A *Luffa* genetic map through an F_2_ population of 130 individuals crossed by two inbred line parents from *Luffa cylindrica* was constructed based on SLAF-seq, and 3701 of the polymorphic markers were mapped to 13 LGs and the map spanned 1518.56 cM with an average distance of 0.41 cM between adjacent markers (Lou et al., 2020). In this study, we successfully generated a genetic map with higher resolution, and 4,673 SNP markers were mapped to 13 LGs with a total length of 2246.74 cM and the average genetic length of LGs was 359.4 cM (**Figure 1**; **Supplementary Table S4**). Most importantly, our map was developed from interspecific crosses between *Luffa acutangula* and *Luffa cylindrica*, in which both phenotypic and genotypic diversity were more abundant. For example, for daily flowering time, approximately a 12-h difference existed between the two cultivars. We believe that our research concerning these traits will assist in breaking interspecific reproductive isolation and promote the utilization of interspecific heterosis in *Luffa* breeding program in the future. In general, we constructed an interspecific high-quality and dense genetic map for *Luffa*, which will be a valuable genomic resource for dissecting numerous *Luffa* traits.

### A genome-wide landscape of genetic controls for multiple agronomic traits in *Luffa*

QTL mapping was performed for the 25 traits linked to maturity, flower size, plant growth and fruit to demonstrate the application of our high-density genetic map. In total, 40 significant QTLs associated with 17 traits were detected across 11 chromosomes except chromosomes 2 and 3. This is so far the largest dataset of genetic controls for agronomic traits in *Luffa* (**Figure 2**; **Supplementary Table S6**). Among the 40 identified loci, nearly four-fifths accounted for > 10% of phenotypic variation, indicating that many genes with major effects were responsible for the genetic controls of these traits.

It was the first research to examine the genetic architecture of the majority of these traits in *Luffa*. Zhao et al. (2021) identified a dwarfism gene *LacDWARF1* on chromosome 5 by combined BSA-Seq and comparative genomics analyses, which was 3 Mb away from our QTL of the 9th internode length. In addition, RNA-Seq revealed that plant hormones, cellular process, cell wall, membrane and stress response were significantly related to the dwarf phenotype (Zhao et al., 2021). Consistent with the findings, candidate genes associated with the length of *Luffa* internodes and branches also exhibited enrichment in these biological processes in our study.

Thereafter, we also identified some important homologous genes of key traits reported in other species. MADS transcriptional regulator genes, *AtSEP3* and *AtSOC1*, were involved in the clock output process of the photoperiodic pathway, where *AtSEP3* is a downstream gene of FLOWERING LOCUS T (FT) and *AtSOC1* is a flowering integrator (Hwan Lee et al., 2012; Adal et al., 2021; Fukazawa et al., 2021). Coincidentally, their homolog genes in *Luffa*, *Lac01g012970* and *Lac01g012980*, were located in our QTL of daily flowering time, which may be the most likely candidate genes for further study. Tomato *SUN* was reported to regulate fruit shape by altering cell division patterns (Wu et al., 2011), and its ortholog in watermelon, *Cla011257*, has also been demonstrated to be associated with fruit shape variation and a deletion of 159 bp in its coding region may cause fruit elongation (Dou et al., 2018). Its *Luffa* ortholog, the calmodulin-binding family gene *Lac10g015060*, targeted by *qYFD1*, was also identified to control young fruit shape. This finding demonstrated that our high-density genetic map was effective in elucidating the genetic basis of complex traits in *Luffa*.

Given all of that, the 40 identified QTLs will lay a solid foundation for further research on the genetic and molecular basis of agronomic traits in *Luffa*.

### *LacCRWN3* regulating the internode diameter variation of *Luffa*

The stem, an important part of plants, performs essential roles in supporting, lodging resistance and nutrient transport to facilitate plant growth and development (Ye et al., 2020). Various molecular mechanisms involved in stem development were confirmed in *Arabidopsis*, including shoot apical meristem development, cyclin, transcript factor and phytohormone pathways responsible for cell organization (Pierre-Jerome et al., 2018). In cucurbit crops, the research on stem diameter has been mainly focused on genetic analysis and QTL mapping (Li et al., 2008; Rao et al., 2021), and there is no relevant study in *Luffa*. In present study, we identified a significant *qID2* associated with main stem internode diameter on chromosome 9, which explained 11% of the phenotypic variation (**Figure 2**; **Supplementary Table S6**). The candidate gene *Lac09g006860* was the only gene harbored in *qID2* and it encoded a CRWN3 protein. CRWN, located in the nucleus, encodes components of putative plant nuclear laminas. Its animal homologous proteins are the major components of the metazoan nuclear lamina (Gruenbaum and Foisner, 2015), which are involved in the maintenance of the chromatin architecture and nuclear shape, gene expression, cell differentiation, and metabolism. Mutations in these proteins cause severe diseases (Eriksson et al., 2003). In recent years, a series of lamina orthologs have also been identified successively from Charophyta to terrestrial plants (Sakamoto, 2020). Typically, *Arabidopsis* has four CRWN proteins, CRWN1 to CRWN4, and they participate in the repair of DNA damage, chromatin organization, transcriptional regulation, and nuclear body formation (Dittmer et al., 2007; Sakamoto, 2020). Their single and double mutants display abnormal nuclear structure and phenotypes (Sakamoto and Takagi, 2013; Wang et al., 2013; Zhao et al., 2016; Wang et al., 2019; Sakamoto, 2020). For example, *crwn1crwn2* showed significantly reduced nuclear size and chromocenter number, CRWN3 played a role in regulating the ABI5 protein level *via* the formation of the degradation body in the nucleus plants, and *crwn1*,*3* had the severe retarded phenotypes, such as lower germination rates, short roots, small leaves, dwarf seedlings, short siliques, and shriveled seeds, whereas *crwn* triple mutants were usually lethal. Meanwhile, severe genomic DNA damage and upregulated expression levels of DNA damage-responsive genes were also detected in the *crwn* mutants lines (Hirakawa and Matsunaga, 2019). Our candidate gene *Lac09g006860* is the only *CRWN3* in cucurbit crops, and it was evolutionarily close to *AtCRWN3* and *AtCRWN2* (**Figure 5**). Sequence alignment and qRT-PCR in the two parental lines showed that, except for SNPs in exons, completely different promoter sequences were detected, and the expression level in thick-stem P93075 were distinctively higher than that in thin-stem S1174 (**Figures 3A–C**). Further natural variation analysis revealed that haplotype P93075-like lines exhibiting significantly thicker stem diameters than those of haplotype S1174-like lines (**Figures 4A, B**). Moreover, unique ABRE motifs related to ABA responses were found in the P93075 allele, which might be consistent with previous reports that *CRWNs* play key roles in ABA-related seed germination and plant development (Zhao et al., 2016; Wang et al., 2019). The results highlighted that *LacCRWN3* might be a positive regulator of *Luffa* internode diameter, and responsible for the internode diameter divergence between *Luffa acutangular* and *Luffa cylindrica* lines.

### Implications for future *Luffa* breeding

Pleiotropic effect is that the QTL responsible for different traits tend to cluster together on a certain region of the chromosome (Verma et al., 2004; Pan et al., 2020b). Our study identified four hotspot regions, and QTLs for strongly correlated traits, including *qFFRN1* and *qFFFN2*, *qOFL1* and *qOFPL1*, were clustered in the same genomic regions, and their increasing effects were all from the *P93075* alleles (**Figure 2**; **Supplementary Table S6**). Meanwhile, some QTLs for traits with weak correlations were also found to be clustered together, such as the QTLs for *qIL* and *qYFPL* on chromosome 7, and *qID* and *qOFPL* on chromosome 8 (**Figure 2**). It is promising that these pleiotropic loci can be utilized to select combinations of various favorable alleles for improving multiple traits through marker-assisted breeding programs of *Luffa* in the future. Moreover, we identified a key candidate gene related to *Luffa* stem development, *LacCRWN3*, whose favorable *P93075-like* allele was significantly positively correlated with stem thickness (**Figures 2**-**4**). We believe that the genetic manipulation of *LacCRWN3* will provide an efficient way to improve lodging resistance and nutrient transport in *Luffa*.

In conclusion, we constructed the first high-density genetic map across interspecies of *Luffa* and identified 40 significant genetic loci controlling different agronomic traits. Except for some key orthologous genes retrieved, a unique gene of the Cucurbitaceae family, *LacCRWN3*, was identified responsible for the *Luffa* internode diameter, and its differential expression profiles associated with the sequence variations in promoter regions might be the main causation for stem thickness divergence in the parental lines. Meanwhile, its two haplotypes, the thin-stem S11174-like and thick-stem P93075-like, provide the molecular evidence for explaining the internode diameter difference among inbred lines. Our findings will provide important genetic information and insights into the dissection of complex traits and breeding improvement in *Luffa*.

## Data availability statement

The data presented in the study are deposited in the CNGB Sequence Archive (CNSA) of China National GeneBank DataBase (CNGBdb) repository, accession numbers: CNP0003546 and CNP0003626.

## Author contributions

HW and JNL conceived and designed the experiments; HW, GZ, JXL, XL, LD, XZ and HG provided support for field phenotyping. LL and YG performed the experiments; LL and GZ provided support for bioinformatics analysis; LL analyzed the data and wrote the manuscript; HW and GZ revised the manuscript. All authors contributed to the article and approved the submitted version.

## Funding

This work was supported by grants from the Laboratory of Lingnan Modern Agriculture Project (NZ2021008), the National Natural Science Foundation of China (31872093 and 31902011), the Science and Technology Program of Guangdong Province (2021A1515012500, 2020A0505020006, 2019A050520002, 201904020012, 2018B020202007 and 2021KJ110), the Science and Technology Program of Guangzhou of China (X20210201020 and 202201011453), China Agriculture Research System of MOF and MARA, Agricultural competitive industry discipline team building project of Guangdong Academy of Agricultural Sciences (202103TD and 202114TD), and the Project of Collaborative Innovation Center of GDAAS(XTXM202203).

## Conflict of interest

The authors declare that the research was conducted in the absence of any commercial or financial relationships that could be construed as a potential conflict of interest.

## Publisher’s note

All claims expressed in this article are solely those of the authors and do not necessarily represent those of their affiliated organizations, or those of the publisher, the editors and the reviewers. Any product that may be evaluated in this article, or claim that may be made by its manufacturer, is not guaranteed or endorsed by the publisher.
