# Supplementary material for: QTL mapping reveals candidate genes for main agronomic traits in Luffa based on a high-resolution genetic map
Source: Front Plant Sci. 2022 Nov 18;13:1069618. doi: 10.3389/fpls.2022.1069618 (PMC9716215; doi:10.3389/fpls.2022.1069618)
Supplement: Supplementary file 1 [file DataSheet_1.docx]

**Supplementary figures**

**
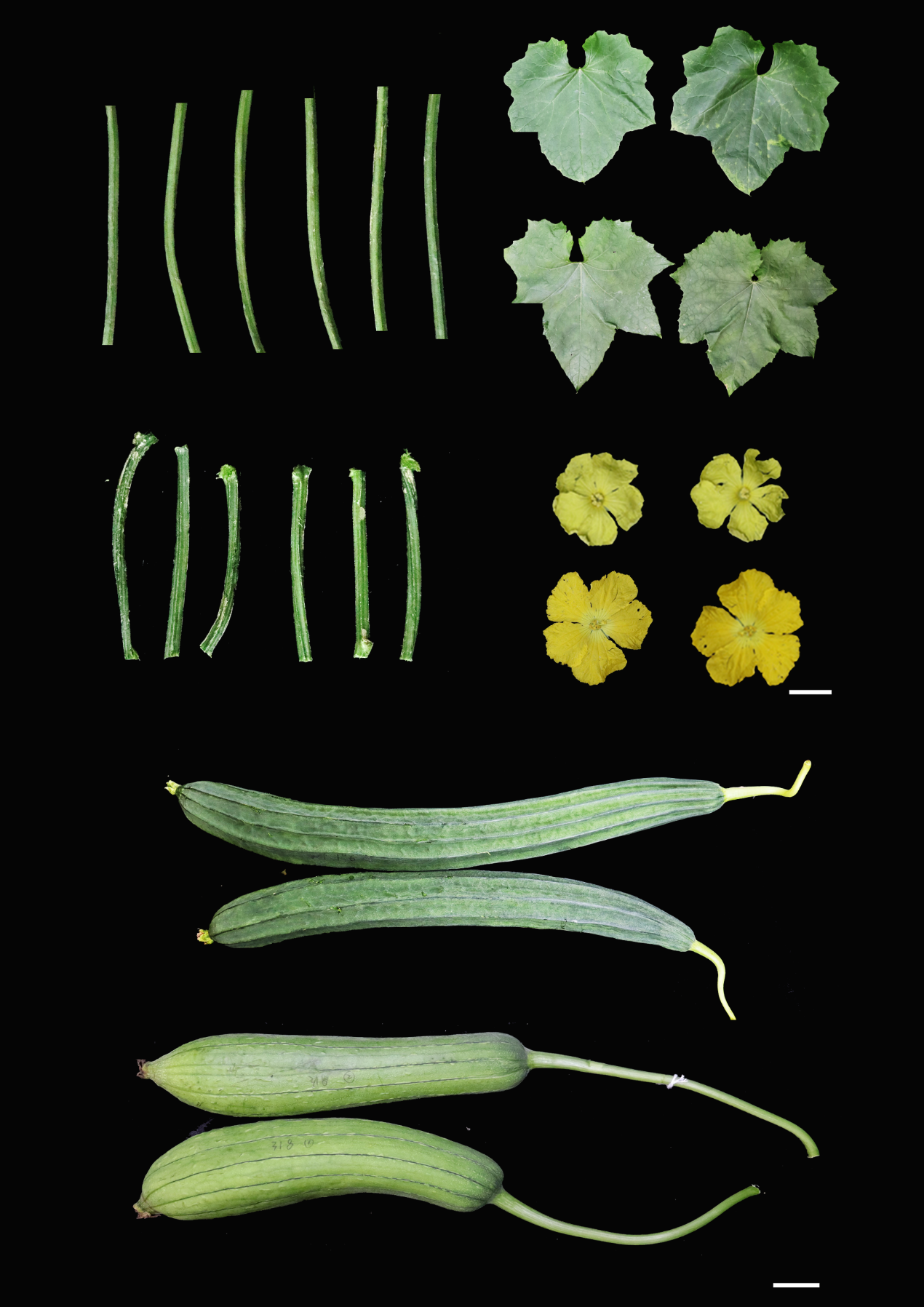
**

**SUPPLEMENTARY FIGURE S1**. The photographs of the two parents. The tissues include stems, leaves, flowers and fruits of S1174 (Above) and P93075 (Below). The bars represent 2 cm for the stems, leaves, flowers, and 4 cm for the fruits, respectively.

**
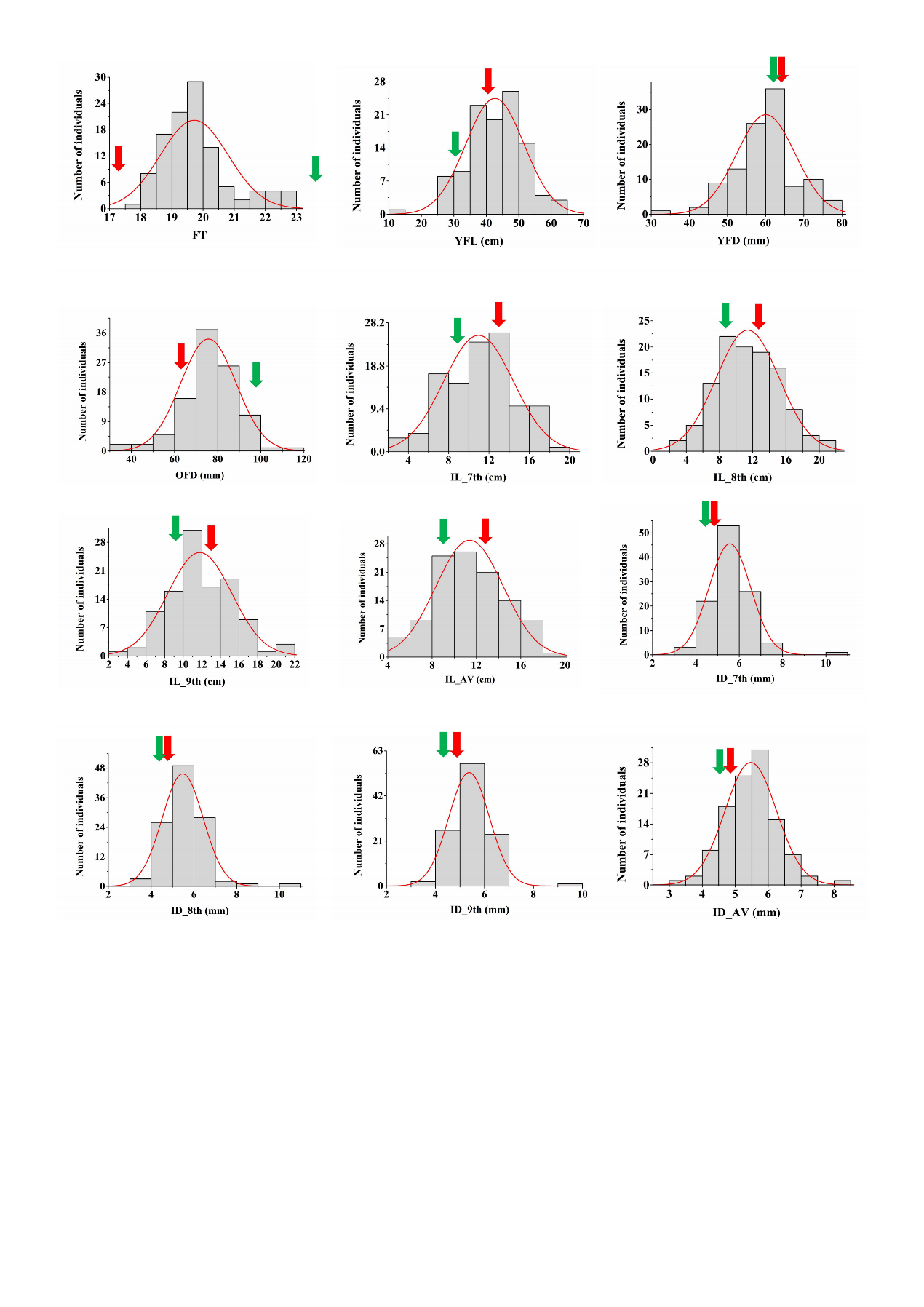
**

**SUPPLEMENTARY FIGURE S2**. Frequency distributions of the key phenotypic traits related to daily flowering time, stem development and fruit shape in the BC_1_ population. Parental values are pointed by red (S1174) and green (P93075) arrows. FT, flowering time; YFL, young fruit length; YFD, young fruit diameter; OFD, old fruit diameter; IL_7th, IL_8th, IL_9th and IL_AV, the respective length of the 7th, 8th and 9th internode and their average value; ID_7th, ID_8th, ID_9th and ID_AV, the respective diameter of the 7th, 8th and 9th internode and their average value.


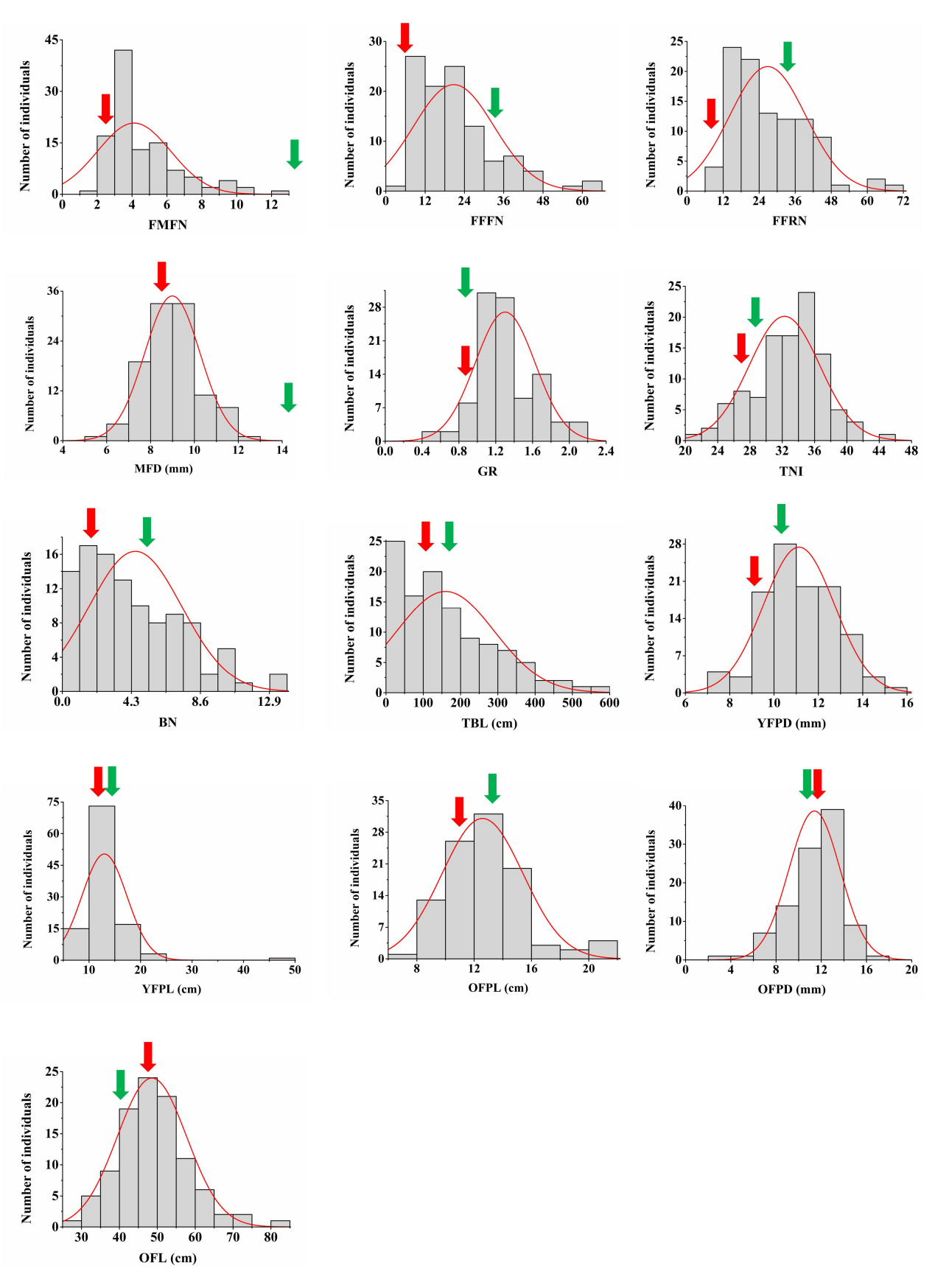


**SUPPLEMENTARY FIGURE S3**. Frequency distributions of other phenotypic traits related to maturity, plant growth and fruit in the BC_1_ population. Parental values are pointed by red (S1174) and green (P93075) arrows. FMFN, first male flower node; FFFN, first female flower node; FFRN, first fruit node; MFD, male flower diameter; GR, growth rate; TNI, total number of internodes; BN, branch number; TBL, total branch length; YFPD, young fruit peduncle diameter; YFPL, young fruit peduncle length; OFPL, old fruit peduncle length; OFPD, old fruit peduncle diameter; OFL, old fruit length.


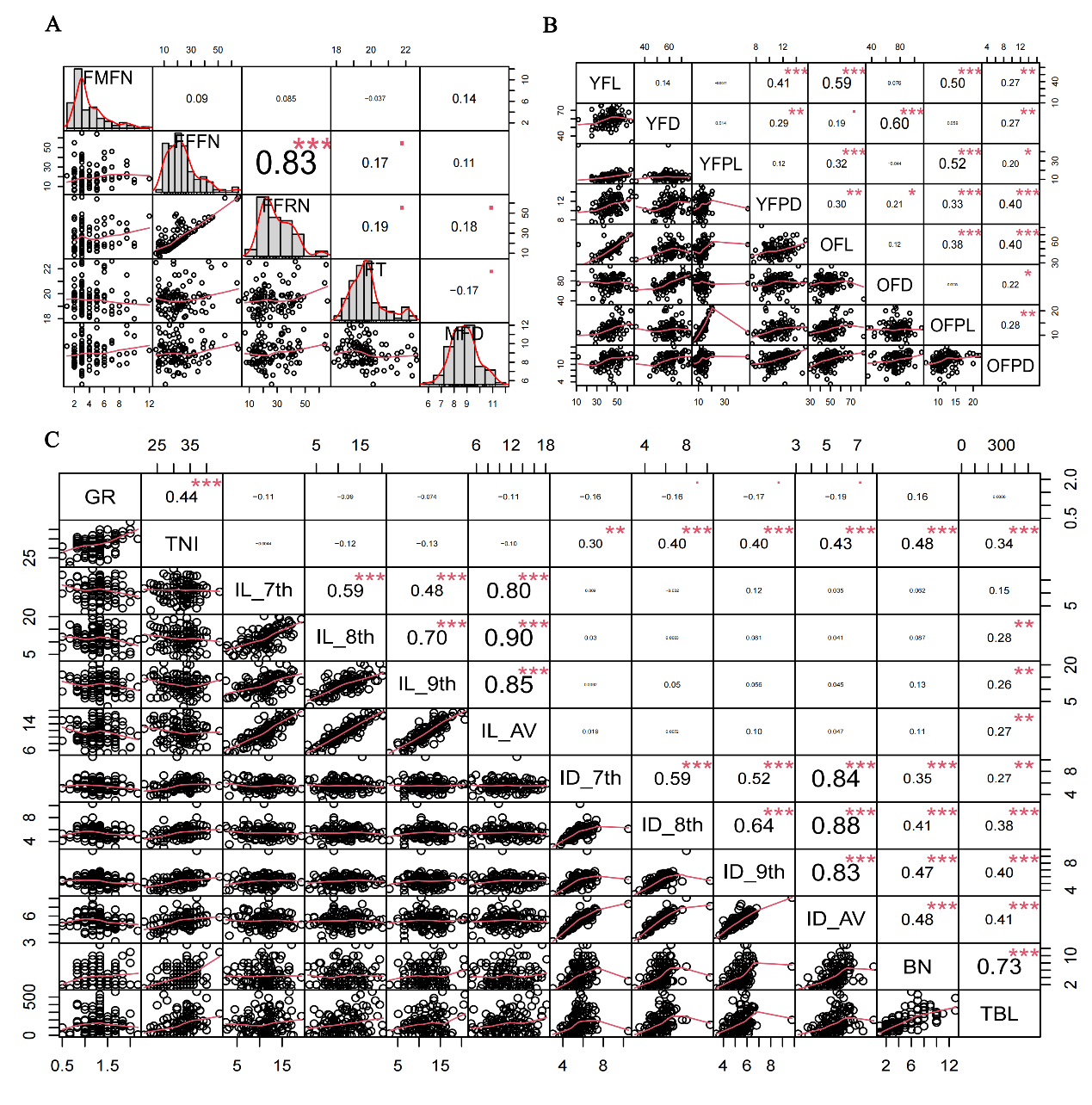


**SUPPLEMENTARY FIGURE S4**. Pairwise correlation analysis of the phenotypic traits in each category. The traits related to maturity and flower size **(A)**, fruit **(B)** and plant growth **(C)**. FMFN, first male flower node; FFFN, first female flower node; FFRN, first fruit node; FT, flowering time; MFD, male flower diameter; YFL, young fruit length; YFD, young fruit diameter; YFPL, young fruit peduncle length; YFPD, young fruit peduncle diameter; OFL, old fruit length; OFD, old fruit diameter; OFPL, old fruit peduncle length; OFPD, old fruit peduncle diameter; GR, growth rate; TNI, total number of internodes; IL_7th, IL_8th, IL_9th and IL_AV, the respective length of the 7th, 8th and 9th internode and their average value; ID_7th, ID_8th, ID_9th and ID_AV, the respective diameter of the 7th, 8th and 9th internode and their average value; BN, branch number; TBL, total branch length. ***, *P* < 0.001; **, *P* < 0.01; *, *P* < 0.05.
